# Measurement Error and Power in Family-Based Extensions to Mendelian Randomization

**DOI:** 10.1007/s10519-025-10236-y

**Published:** 2025-11-03

**Authors:** Luis F. S. Castro-de-Araujo, Madhurbain Singh, Yi Daniel Zhou, Philip Vinh, Hermine H. M. Maes, Brad Verhulst, Conor V. Dolan, Michael C. Neale

**Affiliations:** 1 Virginia Institute for Psychiatric and Behavioral Genetics, Virginia Commonwealth University, 1‑156, P.O. Box 980126, Richmond, VA 23298‑0126 USA; 2 Department of Psychiatry, Austin Health, The University of Melbourne, Melbourne, VIC Australia; 3 Department of Psychiatry and Behavioral Sciences, Texas A&M University, 2900 E 29th Street, Bryan, TX 77802 USA; 4 Department of Biological Psychology, Vrije Universiteit Amsterdam, Transitorium 2B03, Van der Boechorststraat 1, 1081 BT Amsterdam, The Netherlands; 5 Cellular, Molecular and Genetic Medicine, Virginia Commonwealth University, Richmond, USA

**Keywords:** Causality, Pleiotropy, Twin design, Mendelian randomization

## Abstract

Mendelian Randomization (MR) has become an important tool for causal inference in the health sciences. It takes advantage of the random segregation and independent assortment of alleles to control for background confounding factors. In brief, the method works by using genetic variants as instrumental variables, but it depends on the assumption of exclusion restriction, i.e., that the variants affect the outcome exclusively via the exposure variable. Equivalently, the assumption states that there is no horizontal pleiotropy from the variant to the outcome, i.e., no association with the outcome except via the exposure. This assumption is unlikely to hold in nature, so several MR extensions have been developed to increase its robustness against horizontal pleiotropy, though not eliminating the problem entirely (Sanderson et al., in Nat Rev Methods Primer 2:6, 2022). The Direction of Causation (DoC) twin model, which includes information from cross-twin cross-trait correlations to estimate causal paths, was extended with polygenic scores to explicitly model horizontal pleiotropy and a causal path (MR-DoC, Minică et al., in: Behav Genet 48:337–349, 2018). MR-DoC was further extended to accommodate bidirectional causation (MR-DoC2; Castro-de-Araujo et al., in: Behav Genet 53:63–73, 2023). In the present paper, we compared the performance of the DoC, MR-DoC, and MR-DoC2 models to evaluate the effects of phenotypic measurement error, potential unshared (individual-specific) environmental confounding, and statistical power across the three models. It was found that MR-DoC2 is less vulnerable to measurement error than is standard DoC or MR-DoC. The latter two models have biased estimates of causal paths when unshared environmental covariance between exposure and outcome is assumed to be absent.

## Introduction

A long-standing challenge in epidemiology has been to infer causality from correlational data in observational studies. Correlational studies are starting points for exploring the causal associations between variables. However, by themselves, correlations are insufficient to identify causality, due to the potential existence of background confounding and ambiguous direction of causality.

The primary alternative to observational studies is the randomized controlled trial (RCT), in which study participants are randomly allocated to treatment/experimental and control groups. This approach averages the effects of any confounders equally among the groups, so that any difference in the outcome can be attributed to the intervention. However, RCTs are not always feasible. Difficulties may arise due to ethical considerations, e.g., in research including children.

Mendelian randomization (MR) can be used to investigate causality in cases where RCTs are infeasible or unethical. MR is derived from Mendel’s laws of segregation and independent assortment, leveraging the randomization that occurs during meiosis (when genetic information is shuffled between chromosomes and these chromosomes then form the gametes) as a quasi-experimental manipulation (Madole and Harden 2022). Genetic associations with phenotypic exposures identified in large scale genome-wide association studies (GWAS) and meta-analyses, or weighted combinations thereof, are potentially useful instrumental variables (Evans and Davey Smith 2015; Sanderson et al. 2022).

Several key assumptions are involved in causal inference based on MR (Sanderson et al. 2022). First, the association between the genetic variant(s) and the exposure must be strong (typically defined as F-statistic greater than 10), which is known as the *relevance assumption* (Burgess and Thompson 2011). Second, the variant must not be associated with a confounder in the relation between the exposure and outcome, referred to as the *exchangeability assumption.* Third, the effect of a genetic liability change on the exposure variable is the same as an equivalently-sized environmental liability change, i.e., they both generate the same change in the outcome variable. This assumption is known as *gene-environment equivalence* (Howe et al. 2022). It highlights that, unlike in twin-designs, there is no partitioning of additive genetic, shared, and environmental variances in standard MR of unrelated individuals’ data. Fourth, MR is based on the assumption that the instrument’s association with the outcome is completely mediated by the exposure (known as the *exclusion restriction* assumption). In genetic studies, this assumption is known as *no horizontal pleiotropy*. This assumption is unlikely to hold for complex traits, given that GWASs have shown that the same variant often influences multiple traits. Horizontal pleiotropy occurs when the instrument affects the outcome through pathways other than via the exposure variable.

**Fig. 1:**
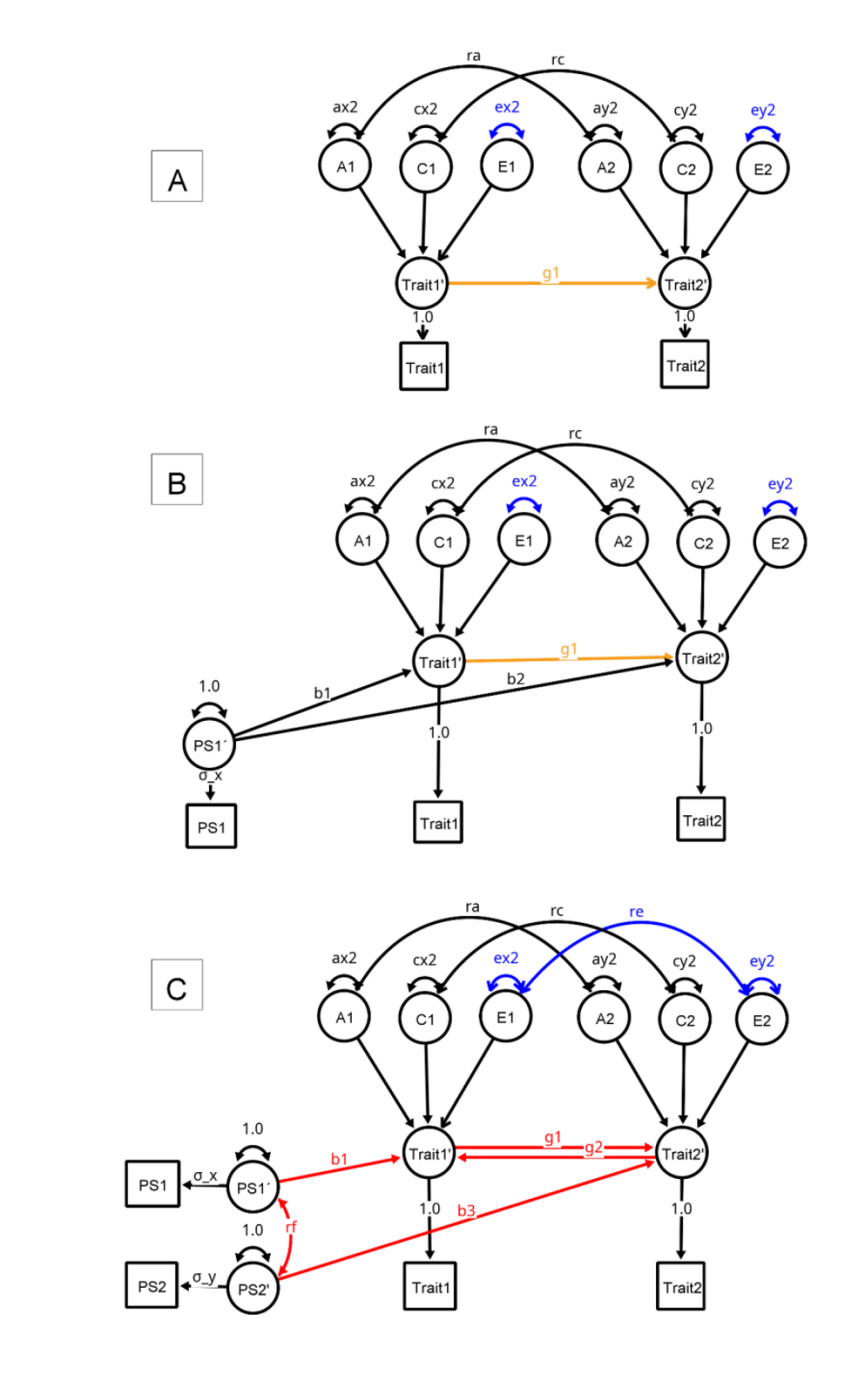
DoC (**A**), MR-DoC (**B**), and MR-DoC2 (**C**) model specifications for a single member of a twin pair. The models include the effects of additive genetic (A), common environment (C) and specific environment (E) factors for both Trait 1 and Trait 2, and their effects may correlate across traits (parameters *ra*, *rc*, and *re*). The genetic cross-twin covariances are multiplied by 1 for MZs and 0.5 for DZs and the shared environmental cross-twin covariances are multiplied by 1 for both MZs and DZs. Path labels in red are important to the model’s power, those susceptible to measurement error in blue, and in orange are those that are both susceptible to measurement error and important to the model’s power. The latent variables Trait1′ and Trait2′ are not required for identification, but are kept to be consistent to the Castro-de-Araujo et al. (2023) paper, to emphasize the scaling solution of PS1′ and PS2′, and to point out that one possible extension of these models is the use of multiple indicators. Models were estimated in variance components, *ra*,* rc*, and *re* were estimated alongside covariances (Color figure online)

Several solutions have been proposed to detect and/or accommodate horizontal pleiotropy in causal inference based on MR (Sanderson et al. 2022). One can use methods that relax this assumption, and only require the instrument strength to be independent of the direct effect of the exposure on the outcome (Bowden et al. 2015), or one can triangulate results from different MR methods to confirm that the strength and direction of the causal signal are consistent over tests (Burgess et al. 2011). Furthermore, estimators based on the mode (Hartwig et al. 2017) and on the weighted median (Bowden et al. 2015) of the instrument effect size estimates were created to address the issue of horizontal pleiotropy. Alternative methods that integrate MR in the twin-design were proposed to address the exclusion restriction assumption (Hwang et al. 2021), notably MR-DoC (Minică et al. 2018), which combines MR with the Direction of Causation (DoC, Fig. 1) twin design (Heath et al. 1993; Duffy and Martin 1994). The MR-DoC model includes a (horizontal) pleiotropic path, accommodating a direct relationship between the instrumental variable and the outcome, and thus allowing for a test of directional horizontal pleiotropy (path *b*_2_, Fig. 1B). A requirement of being able to estimate *b*_2_ in MR-DoC is that it is necessary to fix *re* to zero for identification. MR-DoC was extended to accommodate bidirectional causation in the presence of background confounding (Castro-de-Araujo et al. 2023; MR-DoC2, Fig. 1C) by adding a polygenic score that acts as an instrumental variable for the outcome. The model thus permits estimation of the effects of reverse causation (paths *g*_*2*_, Fig. 1C). Henceforth, we refer to the Minică et al. (2018) model as MR-DoC, and the Castro-de-Araujo et al. (2023) model as MR-DoC2. The list of parameters for each model can be found in Table 1.

**Table 1:** Parameter values in the three factorial designs, with respective total number of cells for each design simulation

θ	Design 1 (DoC)	Design 2 (MR-DoC)	Design 3 (MR-DoC2)
b_1_		$\sqrt{0.025}$, $\sqrt{0.03}$, $\sqrt{0.04}$	$\sqrt{0.025}$, $\sqrt{0.03}$, $\sqrt{0.04}$
b_2_		$\sqrt{0.025}$, $\sqrt{0.03}$, $\sqrt{0.04}$	
b_3_			$\sqrt{0.025}$, $\sqrt{0.03}$, $\sqrt{0.04}$
g_1_	$\sqrt{0.02}$, $\sqrt{0.03}$, $\sqrt{0.04}$	$\sqrt{0.02}$, $\sqrt{0.03}$, $\sqrt{0.04}$	$\sqrt{0.02}$, $\sqrt{0.03}$, $\sqrt{0.04}$
g_2_			$\sqrt{0.02}$, $\sqrt{0.03}$, $\sqrt{0.04}$
ra	0.1,0.2,0.3	0.1,0.2,0.3	0.1,0.2,0.3
rc	0.1,0.2,0.3	0.1,0.2,0.3	0.1,0.2,0.3
re			0.1,0.2,0.3
rf			0.1,0.2,0.5
a_x2_	0.3,0.4,0.5	0.3,0.4,0.5	0.3,0.4,0.5
a_y2_	0.3,0.4,0.5	0.3,0.4,0.5	0.3,0.4,0.5
c_x2_	0.1,0.2,0.3	0.1,0.2,0.3	0.1,0.2,0.3
c_y2_	0.1,0.2,0.3	0.1,0.2,0.3	0.1,0.2,0.3
Code zero estimation	100%	100%	100%
Simulation scenarios	3^7^=2,187	3^9^=19,683	3^12^=531,441

See Fig. 1 for the model specification. Also, e_x2_ was specified as 1 – *a*_*x*2_ – *c*_*x*2_ and e_y2_ as 1 – *a*_*y*2_ – *c*_*y*2_. Parameters σ_x and σ_y are not listed, as they remained unchanged across the designs. Code zero, refers to the percent of estimations that were successful without warnings

The DoC model uses cross-twin cross-trait correlations to extract information on possible causal paths between two phenotypes. However, it is known to have the following limitations. First, differences in reliability of the variables in the model may bias causal inference estimates (Heath et al. 1993; Gillespie et al. 2003). Specifically, the more reliable variable is more likely to be identified as the cause of the less reliable variable (Heath et al. 1993; Duffy and Martin 1994). Second, both the DoC and MR-DOC models require the assumption that the unshared environmental correlation (the parameter *re* in Fig. 1C) is zero in order to estimate the causal path between the exposure and the outcome. This constraint implies that unshared environmental influences are not a source of confounding. Violation of this assumption biases the causal estimates in DoC models (Rasmussen et al. 2019), but it is not a problem for MR-DoC2, which explicitly models this type of confounding. For MR-DoC, it is not clear how much bias incorrectly assuming *re * ≠ 0 would introduce to the estimates of interest, in particular the causal path (*g*_1_), or other paths (Fig. 1B).

The statistical power of the MR-DoC and MR-DoC2 models has been explored in Minică et al. (2018) and in Castro-de-Araujo et al. (2023), respectively. However, a comparison of the power profiles of the three models (i.e., DoC, MR-Doc, and MR-Doc2) is lacking. While all three models focus on causal inference, the models differ with respect to their assumptions. First, the DoC model can accommodate both unidirectional and bidirectional causation, provided that some other parameters are fixed. That is, in addition to the two causal paths, only one of three possible sources (A, C, E) of confounding can be jointly estimated. This implies that of the three correlations, *ra*,* rc*, and *re*, two have to be constrained (to zero, usually, or to another value depending on the hypotheses). In general, a bivariate ACE model is identified with any three of the five possible path coefficients that model the covariance between the two phenotypes (parameters ra, rc, re, *g*_1_ and *g*_2_; Fig. 1) freely estimated (Maes et al. 2021). Second, the MR-DoC model is usually specified with unidirectional causation (as bidirectional causation requires further constraints to the background ACE confounding), and assumes no unshared environmental confounding (*re* = 0, Fig. 1). Third, MR-DoC2 is bidirectional and assumes no direct horizontal pleiotropy (*b*_2_ path in MR-DoC is fixed to zero in MR-DoC2). However, MR-DoC2 does accommodate two sources of indirect horizontal pleiotropy (rf*b_1_ and rf*b_3_ in Fig. 1C), by allowing the instruments to be correlated via *rf*. It also includes vertical pleiotropy via causal effects on the exposure.

In this paper, we compared the statistical power profiles of the three models. We estimated the effects of phenotypic measurement error and unique environmental confounding in DoC and MR-DoC (re ≠ 0). We finally identify situations in which each model performs optimally in terms of power. The outline of this paper is as follows. First, the model specifications will be presented; second, the simulation designs will be explained; third, bias due to measurement error will be tested by introducing unreliable phenotypes; fourth, results from simulations where unique environmental confounding (*re*, in Fig. 1) is fixed to zero when in fact it is present in the data generation process will be presented. Finally, the statistical power of the three models will be reported.

## Methods

### Model Specification

Specifications of the three models were reported in the original papers: DoC (Heath et al. 1993; Neale and Cardon 1992), MR-DoC (Minică et al. 2018), and MR-DoC2 (Castro-de-Araujo et al. 2023). All three models share the classical twin-design’s partitioning variance into additive genetic (A), shared environmental (C), and unique environmental (E) components (Fig. 1). The models are bivariate: Trait1 and Trait2 are the phenotypes, and they are specified such that zero, one or two causal paths may exist between the two traits (g_1_ or g_2_, or both). The classic twin model is in Panel A of Fig. 1, known as the Direction of Causation model (Heath et al. 1993), which is nested in both the MR-DoC and MR-DoC2 models. The specification of the DoC model tested here includes covariation/confounding due to additive genetic factors (*ra*) and to shared environmental factors (*rc*). MR-DoC is not nested in MR-DoC2 due to the presence of constraints needed for identification (the presence of direct horizontal pleiotropy paths from PS1 to Trait 2’ in MR-DoC). These models were specified in OpenMx code using matrix algebra and the variance component approach (Verhulst et al. 2019). Note that in previous papers the models were specified using path coefficients (Minică et al. 2018; Castro-de-Araujo et al. 2023). Notably, in the present specification the variances are estimated, and the paths are fixed, whereas in previous specifications the variances were fixed and paths estimated. In this version, variances are allowed to be negative, which is known to result in better calibrated type I error rates (Verhulst et al. 2019). However, the ra, rc, and re correlations are estimated, and these correlations are reported in the diagram and all plots. This approach is consistent with recent work in our group (Maes et al. 2022; Castro-de-Araujo et al. 2024). The code for each model is publicly available as a function in the *umx* R package (Bates et al. 2019).

### Simulation Designs

We conducted simulation studies to compare the DoC, MR-DoC and MR-DoC2 models, and to investigate known limitations of the DoC and MR-DoC twin models. We addressed three issues: the effect of unmodeled phenotypic measurement error on parameter estimates; the effect of misspecification of models with respect to the *re* parameter (*incorrectly constrained re* = 0); and power, by assessing the associations between the models’ parameters and the non-centrality parameter in tests of null-hypotheses in each model.

We used exact data simulation to generate raw data given a population covariance matrix. Using this method, we ensure that the covariance matrix of the generated raw data exactly equals the population covariance matrix. This equality means that when fitting the true model (given that it is identified) the parameter estimates match the population values exactly. Consequently, a hypothesis test based on the likelihood ratio test (e.g., fixing a parameter to zero), can be regarded as a non-centrality parameter, which we can use in power calculations (van der Sluis et al. 2008). The method comprises the following five steps. (1) Choose values for the parameters in the model of interest. (2) Simulate multivariate normally distributed raw data based on the model’s expected covariance matrices and means of the monozygotic (MZ) and dizygotic (DZ) twins. To this end, we used the function *mvrnorm()* in the R library MASS with MZ and DZ sample sizes at 1000 pairs (Venables et al. 2002). The option empirical = TRUE was used to remove sampling variation in the simulated samples’ means, variances and covariances. (3) Fit the true model using maximum likelihood estimation, thus recovering the true parameter values. (4) Fit a false model by imposing the constraint(s) of interest (e.g., fixing a parameter to zero). (5) Extract the non-centrality parameter (NCP), which equals the difference in minus twice the log-likelihood of the models fitted in steps 3 and 4, and use it to calculate the power to reject the parameter of interest given a Type I error rate of 0.05. (6) Bias is obtained by subtracting the average true values in a factor by the average of estimated values of a parameter, then plotted in bar plots.

In what follows, we present a total of five simulations, with a combination of factorial designs and data generation processes. The designs are listed in Table 1, in which parameters are featured as design factors, and their values as the factor levels. The values chosen were comparable to those used in a previous publication (Castro-de-Araujo et al. 2023), which attempted to model phenotypes with heritability between 0.3 and 0.5 and strong instruments (b_1_, and b_3_) between 0.16 and 0.20. All simulation designs included multivariate models with A, C, and E variances. In the interest of conciseness, factor levels (values of parameters) are equal across designs. Although the specification used was of the variance components type, we calculated the correlations ra, rc, and re with matrix algebra to avoid mixing covariance components and correlations. Finally, the number of parameters in each design depends on the number of parameters in each of the models (DoC, MR-DoC, and MR-DoC2), thus Design 1 has 9 parameters (*g*_1_,*a*_*x*2_, *c*_*x*2_, *e*_*x*2_, *a*_*y*2_, *c*_*y*2_, *e*_*y*2_, *ra*, and *rc*); Design 2, has 11 parameters (*g*_*1*_, *b*_1_, *b*_2_, *a*_*x*2_, *c*_*x*2_, *e*_*x*2_, *a*_*y*2_, *c*_*y*2_, *e*_*y*2_, *ra*, and *rc*) and Design 3 has 14 parameters (*g*_*1*_, *g*_2_, *b*_*1*_, *b*_3_, *rf*,* a*_*x*2_, *c*_*x*2_, *e*_*x*2_, *a*_*y*2_, *c*_*y*2_, *e*_*y*2_, *ra*,* rc*, and *rc*).

In the first set of simulations, the models were fitted to data generated from the same design, i.e. Design 1 for DoC, Design 2 for MR-DoC and Design 3 for MR-DoC2. Simulations 1 and 2 were designed to evaluate the effects of unreliable phenotypes and the effect of unshared environmental confounding on bias for all three models (Table 1, Designs 1, 2 and 3). The next set of simulations fitted each model to data generated exactly according to the MR-DoC2 covariance matrix generated by the parameter values in Table 1, Design 3. The reasoning behind generating data with the more complex model, was to facilitate comparisons between the three models using the least restrictive (and possibly the most realistic) model to generate the data. Simulations 1 and 2 were repeated with the new data generation process. The final simulation evaluated the power of the three models to reject that the causal parameter is zero.

#### Simulation 1: Unreliable Phenotypes

**Fig. 2:**
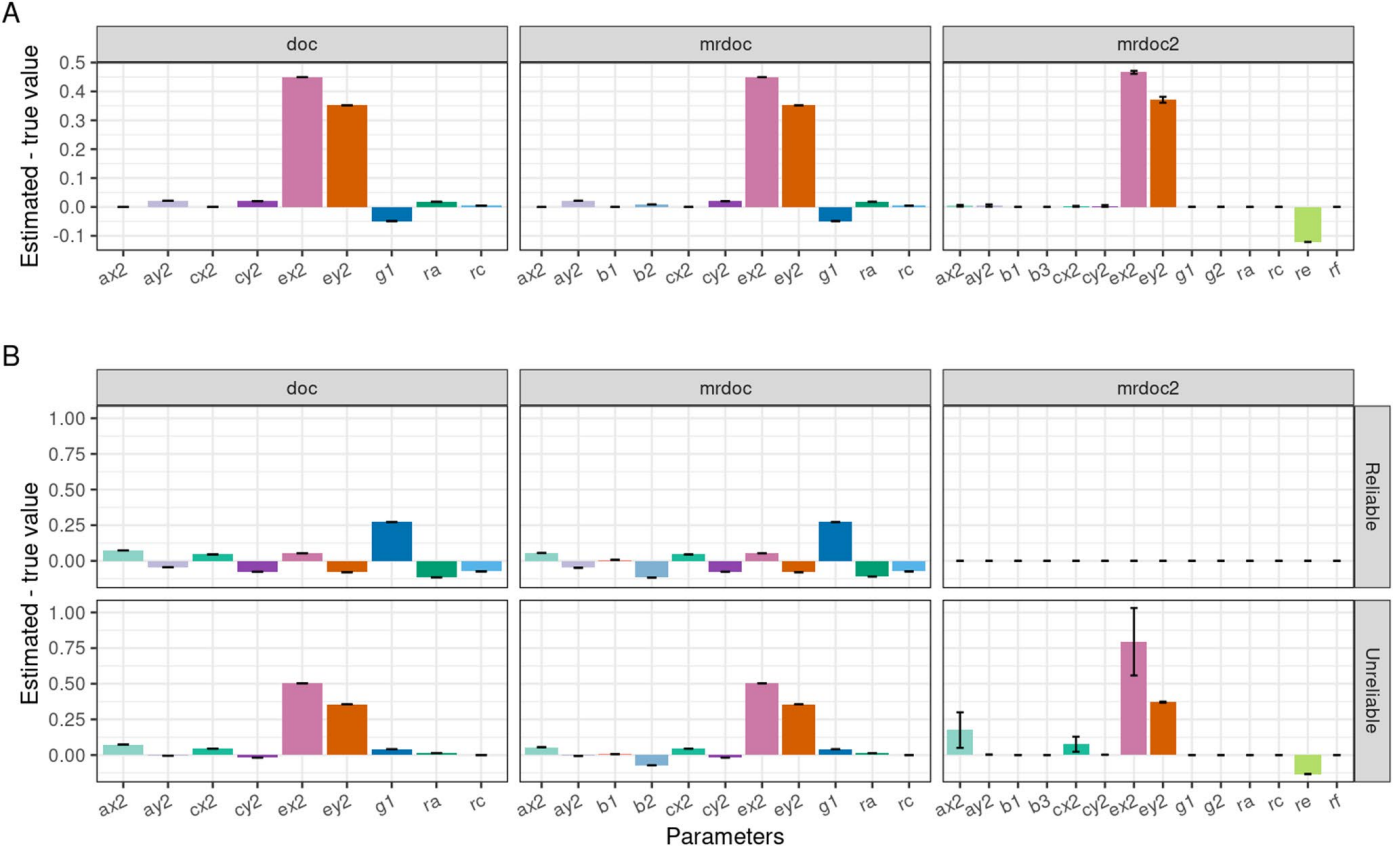
Simulation results of testing bias due to measurement error in DoC, MR-DoC, and MR-DoC2. For each estimated parameter on the x-axis, the mean difference between the true parameter values and the parameter estimates in the exact data simulations is plotted. Reliability was set at 90% in the exposure (Trait 1) and 70% in the outcome (Trait 2) in an exact data simulation (Designs 1, 2, and 3; Table 1). **A** Simulation when models were fitted to data generated with the specific model being tested. **B** Data generation process is MR-DoC2

We first assessed the effect of unreliability in the phenotypic measurements on the parameter estimates. The reliabilities of the phenotypes were set to reflect the known shortcoming of the DoC model, in which the more reliable phenotype is more likely to be identified as the cause of the less reliable phenotype, thus reliability for the exposure was set to 0.90 and that of the outcome to 0.70. This was done by specifying a measurement error term at the phenotypic level with values fixed at 0.1 and 0.3 in Trait 1 and Trait 2, respectively. The measurement error was modelled so that it is not part of the transmissible variance between the two traits. Essentially, the exposure and outcome phenotypes were replaced by latent variables, between which the causation was modelled. The latent variables caused their respective observed variables, with a regression coefficient equal to the square root of the reliability. The observed variables had residual variance set to one minus the reliability. This new covariance structure was then used as data to fit the models. Bias stemming from the unreliability was calculated as the mean difference between the true parameter values and the parameter estimates averaged across the exact data simulations (Fig. 2A).

## Results

### Bias Due to Measurement Error

To assess the impact of phenotypic measurement error, we introduced unreliability to both the exposure (10%; i.e., reliability 0.9) and the outcome (30%; i.e., reliability 0.7). The results obtained from the simulations are shown in Fig. 2. In DoC and MR-DoC the causal path (*g*1) was underestimated given unreliable phenotypic measurement, while estimates of the unique environment were biased upward, proportional to the amount of unreliability (Fig. 2A). Measurement error did not affect the causal path estimates (*g*1, *g*2) in MR-DoC2. Therefore, MR-DoC2 was more robust than MR-DoC when the proportion of measurement error differs between the phenotypes.

When the data were generated using the MR-DoC2 model (Fig. 2B), we found similar bias levels in DoC and MR-DoC. The absence of the paths from MR-DoC2 in MR-DoC (*re*, *rf*, *b3*) and DoC (*b1*, *b*_2_), resulted in widespread bias in the models’ estimates with notable overestimation of *g*1 with or without measurement error. Estimates of the unique environment variance were biased upward in the presence of measurement error (unreliable phenotypes).

### Bias Due to Environmental Confounding

**Fig. 3:**
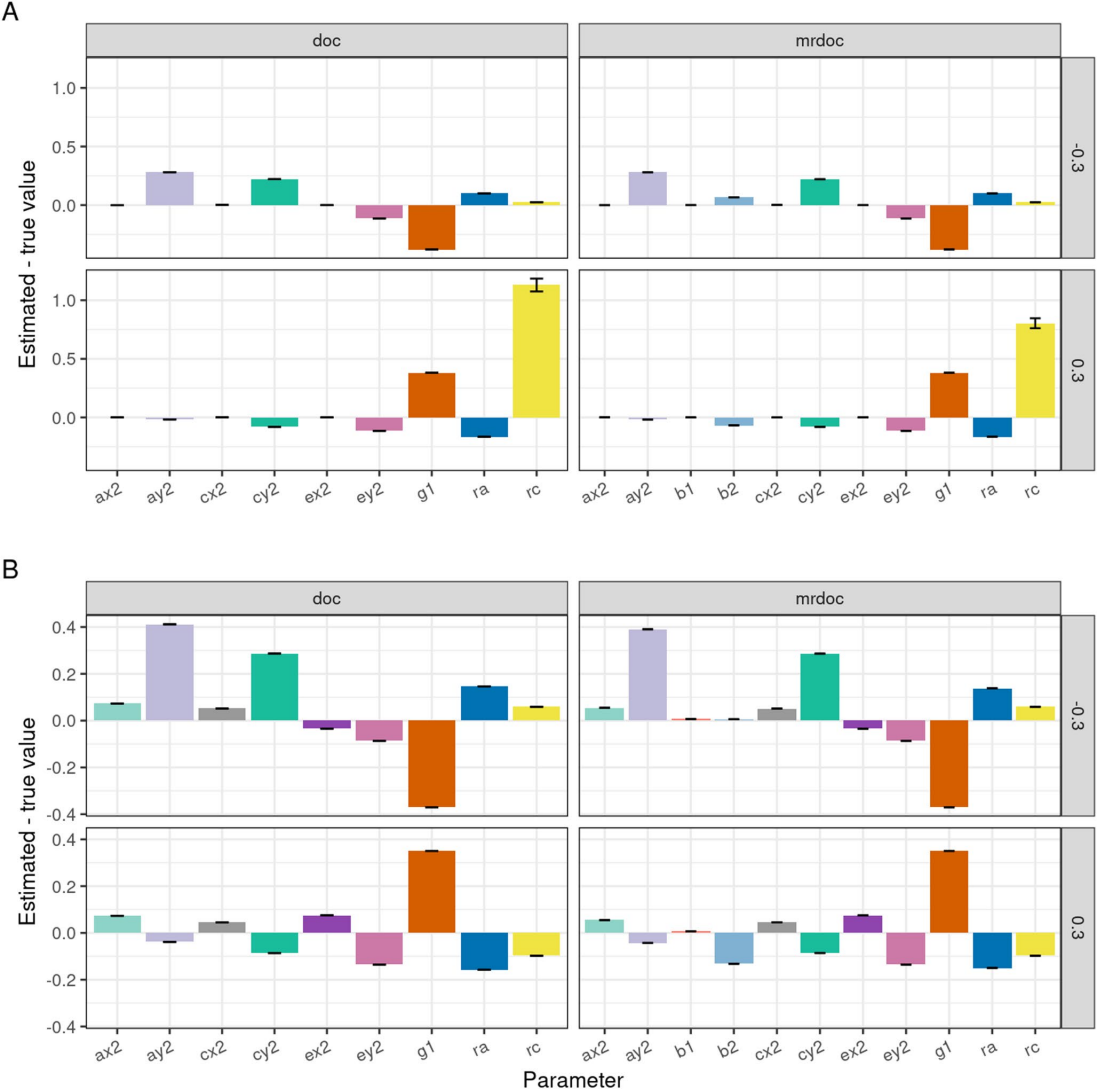
Simulation results testing bias due misspecification of the unique environmental confounding (*re*) parameter. **A** refers to the simulations when models were fitted to data generated with the specific model being tested. **B** refers to the simulations using MR-DoC2 as the data generating process (Design 3, Table 1). Top facets: exact data generation of *re* = + 0.3. Bottom facets: exact data generation for *re* = − 0.3. MR-DoC2 presented no bias in these tests

Results of simulating the effect of the misspecification of unique environmental confounding (*re*) are presented in Fig. 3A, B. If *re* was truly positive (+ 0.3), the specification *re* = 0 resulted in an overestimation of the causal parameter *g*1 in both DoC and MR-DoC (Fig. 3A, bottom facet). Conversely, *g*1 was underestimated when *re* was truly negative (− 0.3) but assumed to be zero (Fig. 3A, top facet). Thus, when DoC and MR-DoC models were fitted to data generated by MR-DoC2, the bias in the parameter estimates was widespread (Fig. 3B). By contrast, the causal (*g*1, *g*2) and pleiotropic (b1, b3) paths remain unbiased in the MR-DoC2 simulation (not shown).

### The Effect of Each Model Parameter on Power

**Fig. 4:**
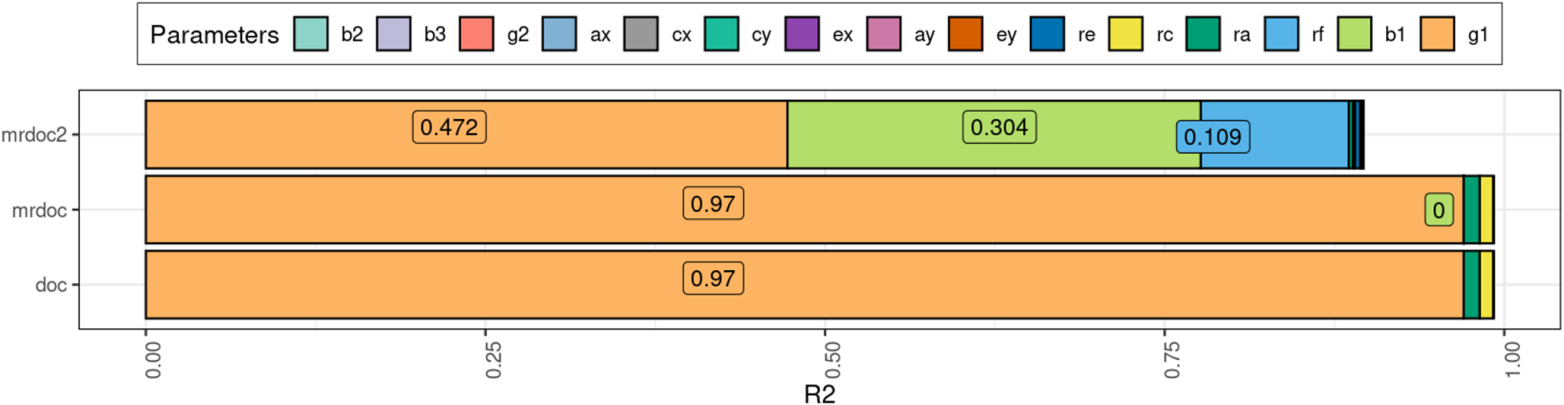
Simulation results from estimating statistical power of DoC, MR-DoC and MR-DoC2 to reject the hypothesis of *g*1 = 0. Data were standardized. Stacked bars represent regression coefficients of the non-centrality parameter (NCP) as a function of model parameter values. The total R^2^ for the DoC model was 0.99, 0.99 for MR-DoC, and 0.90 for MR-DoC2

We compared the statistical power profiles of each model. For this step, the models were fitted to data generated with the specific model being tested, i.e. Design 1 for DoC, Design 2 for MR-DoC and Design 3 for MR-DoC2. MR-DoC2 is a bidirectional model, but here we focus only on the power to reject the hypothesis that *g*1 = 0, at an alpha level of 0.05. Data were standardized with cov2cor() in the expected covariance matrix for MZs and DZs before estimation. The NCPs from this power test were regressed on the parameter values, and the coefficients for each regression were plotted as a stacked bar plot in Fig. 4. The total R^2^, the proportion of NCP variance explained by the parameters equalled 0.99 in the DoC model, 0.99 in the MR-DoC model, and 0.90 in the MR-DoC2 model. The longer the bars of a given parameter, the more NCP variance the parameter explains. We found that *g*1 had the largest effect on DoC and MR-DoC power to detect *g*1, and that, *b1* and *rf* also had large effects on the MR-DoC2 power. Also, *ra* and *rc* had small, but noticeable, effects on the power in the DoC and MR-DoC models. Note that, in MR-DoC, the path from the instrument to the exposure (b1) did not contribute to the power of the model. This means that the variance explained by the instrument does not affect the power to reject the hypothesis that *g*1 = 0 at the 0.05 significance level in MR-DoC. Furthermore, all power tests performed were of the hypothesis *g*1 = 0 to make the results more comparable between models. However, it is also possible to perform a 2df power test in MR-DoC2 dropping both *g*1 and *g*2.

## Discussion

We presented a series of simulations that address issues regarding: (1) measurement error, (2) misspecification of non-shared environmental confounding, and (3) the statistical power of each parameter on three models: DoC, MR-DoC, and MR-DoC2. We found that the models differed in how they are affected by issues 1 and 2, and in the role played by the instrumental variable(s) when comparing MR-DoC with MR-DoC2. The estimates of the causal path (*g*1) were biased in DoC and MR-DoC models when there was measurement error of the phenotypes, or when unique environmental confounding (*re*) was misspecified as equal to zero. We also found that the power profiles differed between the models. For MR-DoC2, all three of *b*1, * g*1 and *rf* had large effects on power to reject the hypothesis that *g*1 = 0, whereas in the DoC and MR-DoC the only such parameter was *g*1 (Fig. 4). We color-coded Fig. 1 to illustrate these results; the paths marked in blue have biased estimates in the case of misspecification of *re*, the ones in red contribute relatively substantially to the NCP variance (power), and the ones in orange are both important to power and biased by *re* misspecification.

When evaluating the power profile of MR-DoC (estimating *b*_2_ and fixing *re = 0*), we found that the instrument strength does not explain any variance on the NCP to reject the false hypothesis of no causation. In other words, there is no requirement of a strong instrument in MR-DoC’s case. MR-DoC explicitly includes horizontal pleiotropy in the parameter *b*_2_. It is therefore a model that addresses this problem directly, allowing causal inference adjusted for the presence of horizontal pleiotropy. To identify the *b*_2_ parameter it is necessary to constrain other parameters, like *rc* or *re*. It should be noted, however, that *b*_2_ was slightly biased by unmodeled non-zero *re* (Fig. 3).

These MR-DoC characteristics (no effect of the instrument in explaining the NCP variance, and the biased *b*_2_ in the presence of *re*) raise the question of what MR-DoC provides over and above the classic DoC causal estimation. Figures 2, 3 and 4 shows that MR-DoC behaves very similarly to DoC in terms of biases and power. MR-DoC would be suitable in cases where *re* has a previously established accurate estimate and can be fixed, allowing for the estimation of the horizontal pleiotropy path (*b*_2_). However, such estimates are unlikely to be available and accurate, so an alternative is needed. One practical approach would be to use a sensitivity analysis, fixing *re* to a range of values and reporting the obtained *b*_2_ for the range.

In summary, there are two ways of thinking about the effect of the no *re* assumption on the bias present in DoC and MR-DoC models. One way is to consider each variance component a latent instrument. E variance incorporates measurement error in all three models tested, however in DoC and MR-DoC, the absence of *re* has the effect of turning the E variances into latent instruments (the PRSs are observed instruments). Since measurement error is subsumed in the E variance of DoC and MR-DoC, this results in bias in the causal estimates. A second way to think about these biases is that the lack of *re* introduces endogeneity between exposure and outcome, correlated errors between Trait 1 and Trait 2. In this situation, the causal estimates will be biased in a way similar to what happens in linear regressions when there are correlated errors in the data generating process (Bollen 2012; Maydeu-Olivares et al. 2019) and b_1_ will not act as an instrumental variable (as seen in Fig. 4 in MR-DoC). In MR-DoC2, because *re* is specified, these effects do not occur.

Parameter estimates from the MR-DoC2 model were consistently the least biased in all tests performed. The assumption of *re* = 0 and absence of partly pleiotropic pathways like rf*b1 or rf*b3 in MR-DoC and in DoC biased the causal path estimate (*g*1). Another strength of MR-DoC2 is its feedback loop structure, thereby allowing inference regarding bidirectional causation. Feedback loops are frequently found in nature, and most current MR methods (often based on linear regression) can only evaluate this type of relationship by running the test twice, one exception being Darrous et al. (2021), changing the instrument in each direction (Timpson et al. 2011; Hwang and Evans 2024).

The tests presented also revealed an important aspect of model comparisons in Structural Equation Modeling (SEM). Due to the non-independence of model parameters, a change to one of them will usually result in changes to the other parameters (Figs. 2 and 3). For consistency with recent publications (Verhulst et al. 2019; Maes et al. 2022), we converted all three models to the variance component style. The variance component style (as opposed to the more traditional reticular action model, RAM, specification) has a much better calibrated Type I error, especially in the multivariate case (Verhulst et al. 2019).

This study should be interpreted in the light of the following limitations. The bias analysis and the results of the power analyses of these SEM models serve only as an aid to understanding how biases may arise and the power in specific scenarios considered. Changes to a single parameter in these models leads to changes in most other paths, making comparisons and interpretation not straightforward. For example, setting *g*2 = 0 in the power test revealed that *b*3, *rf*, and *g*2 were influential to the NCP variance (not shown).

The models presented here are notable because they overcome serious limitations inherent in classical MR. MR-DoC (with the *b*_2_ parameter freely estimated and *re* fixed at zero) does not require a strong instrument, and bidirectional causal inference is possible with cross-sectional data. MR-DoC includes direct horizontal pleiotropy, as does MR-DoC2, which includes indirect horizontal pleiotropy. Furthermore, the models can be extended to relatives of any type (such as siblings, for example) and when such data are available, these models offer interesting new possibilities like true bidirectional causal inference or being able to test for causality while controlling for horizontal pleiotropy.

## Data Availability

Data sharing is not applicable to this article as no datasets were generated or analyzed during the current study.

## Code Availability

Code is available in a repository for replication (https://github.com/lf-araujo/2024-reliability-mrdoc*)* .
